# Facilitating Teamwork With Minimal Interaction via a Mesh-Based Communication Device in a Clinical Nursing Setting: Intervention Study

**DOI:** 10.2196/85599

**Published:** 2026-08-05

**Authors:** Yujiro Matsuishi, Masakazu Hirokawa, Yoshiaki Inoue, Kenji Suzuki

**Affiliations:** 1 Science and Engineering, Women’s Health Nursing and Midwifery, Institute of Medicine, University of Tsukuba, 1-1-1 Tennodai, Tsukuba, Ibaraki, 305-8577, Japan, 81-29-853-2111; 2 Artificial Intelligence Laboratory, University of Tsukuba, Tsukuba, Ibaraki, Japan; 3 Department of Emergency and Critical Care Medicine, Institute of Medicine, University of Tsukuba, Tsukuba, Ibaraki, Japan

**Keywords:** nursing communication, teamwork, digital health, response time, mobile apps

## Abstract

**Background:**

The prevention of medical errors depends on effective communication and teamwork between nurses, yet traditional nurse call systems create inefficiencies that result in higher staff workloads. Further research is needed to evaluate how digital health care technologies affect nursing workflows and whether they contribute to staff workload.

**Objective:**

This study aimed to evaluate whether a smartphone app using Bluetooth Low Energy (BLE) Mesh–based signal communication could enhance nurse-to-nurse communication, decrease response times, and improve teamwork as measured by the TeamSTEPPS Teamwork Perceptions Questionnaire (T-TPQ).

**Methods:**

We conducted a between-subjects (independent groups) interventional study on an acute care ward, with 3 intervention days (BLE Mesh app use) and 3 nonintervention days administered on alternating days. Thirty nurses (15 per condition) participated, with no nurse contributing observations to both conditions in the final analysis. Response times were tracked, and the T-TPQ was used to evaluate Situation Monitoring and Mutual Support perceptions at the end of each shift.

**Results:**

The app shortened the total response duration from a per-nurse median of 38.5 (IQR 31.0-58.5) seconds in the control condition to 19.5 (IQR 18.5-23.5) seconds in the intervention condition (n=15 per condition; Mann-Whitney *U* test=217.5; *P*<.001; rank-biserial *r*=–0.93). The app reduced the time it took for staff to confirm nursing actions, search for items, and request care assistance. A total of 40 nurses participated, generating 48 questionnaire responses; after exclusions, 30 nurses (15 per condition) were analyzed. T-TPQ confirmed significant improvements in Situation Monitoring (3.04 vs 3.39; *P*=.001) and Mutual Support (3.01 vs 3.31; *P*=.003). Post hoc power ranged from 87% to 92% (Cohen *d*>1.0), supporting the interpretability of these results.

**Conclusions:**

BLE Mesh technology reduces basic signal communication delays, which shortens nurse response times and enhances team performance through improved Situation Monitoring and Mutual Support, according to our research. The study demonstrates how this technology delivers functional benefits that enhance nursing communication to produce superior team-based care outcomes.

## Introduction

Effective communication and robust teamwork are foundational pillars of high-quality health care delivery, particularly within nursing practice. The intricate nature of patient care necessitates continuous, efficient, and accurate information exchange among health care professionals to ensure comprehensive and holistic patient outcomes [1]. This “professional communication” is paramount for facilitating information sharing, enhancing the appropriateness of medical interventions, and ultimately improving patient safety [2]. Conversely, communication failures represent a pervasive and critical issue in modern health care, frequently leading to medical errors, adverse events, and even preventable patient mortality [1-3]. Indeed, ineffective communication often creates obstacles to information flow among health care teams, a lack of teamwork, organizational inefficiencies, and decreased staff morale. Notably, poor communication has been identified as the sole cause in 13.2% of patient safety incidents and a contributing factor in 24.0% of incidents [4]. Factors such as hierarchical structures, cultural differences, or even disruptive behaviors in the health care environment can hinder effective communication, highlighting the need for improvements through organizational commitment, training, and standardized policies [2]. This underscores the urgent necessity for innovative solutions to enhance communication and collaboration within health care teams.

Despite the recognized importance of effective communication, nurses frequently encounter significant challenges that impede seamless information transfer and mutual support. For example, traditional nurse call systems are predominantly static and “place-oriented,” confining patients to fixed locations for initiating calls and lacking crucial contextual information regarding patient risk factors or staff availability [2,5]. This leads to considerable inefficiencies, including unnecessary interruptions for nurses, wasted time spent locating staff (≈10% of nursing time is spent “looking for someone”), and inappropriate call routing, where highly skilled registered nurses are often engaged in tasks outside their professional scope [2,5]. Such situations can increase nurses’ workload and potentially diminish the quality of care.

Furthermore, while digital technologies are increasingly integrated into nursing practice with the aim of improving efficiency and communication, their impact is not always positive. The adoption of information and communication technologies, such as smartphones and wearable devices, indicates a tendency for nurses to use these tools for information retrieval and communication with health care team members. However, their overall impact on nurses’ perceived workload has been complex, often proving “mainly negative” [6-8]. This suggests that simply introducing technology, without careful consideration of workflow integration and cognitive load, can inadvertently exacerbate existing burdens [5]. Barriers to the adoption of digital health technologies include infrastructural and technical issues, psychological and personal concerns, and worries about increased working hours and workload. Conversely, training, perceived usefulness, government incentives, and user involvement in development have been identified as facilitators for adoption [5].

Bluetooth Low Energy (BLE) Mesh is a communication solution characterized by its flexibility, reliability, and low power consumption. It offers the advantage of enhanced network coverage and internetwork connectivity, which is especially beneficial in complex hospital environments [8]. While BLE has seen applications in the medical field, such as for contact tracing and patient monitoring systems [8,9], research specifically focused on leveraging this technology for dynamic, peer-to-peer communication and mutual support among nurses, with an emphasis on concretely reducing workload and improving communication efficiency, largely remains unexplored. This app is characterized by simple signals conveying mutual support among nurses. Therefore, to address these significant gaps and challenges, this study developed and validated a novel, simple signal communication app using BLE Mesh network technology.

## Methods

### Study Design

This study used a between-subjects (independent groups) interventional design, comparing nurse response times (RTs) and teamwork perceptions between an intervention condition (using the BLE Mesh app) and a nonintervention condition (without the app), administered on alternating days. Different nurses participated in each condition; in the final analysis, no individual nurse contributed observations to both groups.

### Intervention Design

This research comprises 2 distinct phases: intervention and nonintervention days administered on alternating days, beginning with a nonintervention day. The study included 3 app-use days alongside 3 app-free days for a complete observation period of 6 days.

### Participants

Each shift on the ward was staffed by approximately 8 room-based nurses, including the charge nurse, who shared primary responsibility for direct patient care. Because nurses were assigned to shifts according to the standard ward roster rather than being fixed across the study period, a total of 40 unique nurses participated across the 6 study days, generating 24 questionnaire responses per condition (48 responses in total). Different nurses contributed to the intervention and nonintervention conditions, and those who happened to provide responses under both conditions were excluded from the final analysis (see “Demographic Data” section).

### Experimental App

This study developed a prototype smartphone app as research support for social signal communication between nurses. Through its core technology, the app builds a mesh network using BLE to allow real-time communication between multiple devices. Users can access the app through smartphones running Android OS (Android 11 or later) with dynamic mesh network configurations. The study used 8 Google Pixel 4a devices running Android 13. The app functions through 2 different call systems that distinguish between critical and nonurgent assistance needs: green call and red call. In green call, the function is intended for nonemergency help requests. The app communicates both call types and sender names to the other nurses’ terminals when a user initiates a call. The call is cleared when any recipient presses the designated response button; a confirmation notice is then displayed for 20 seconds on the sender’s terminal (10 s on the other terminals). In red call, the function serves as an urgent help request system. The notification persists until 2 responders answer the call or the initiator manually ends the alert.

The app was developed as a research prototype by the study team. The Android app was built using Android Studio (Google LLC) and implements a custom application-layer BLE relay protocol, enabling multihop, infrastructure-free, device-to-device communication. Message delivery was subjectively immediate in prestudy testing, although formal latency measurements were not performed. The prototype underwent iterative usability testing by the research team prior to deployment.

### Experimental Procedure and Data Collection

The nurses who participated in the study received Android devices with the app they needed to complete for this research. The data collection process included both days with app use and days without app use according to the following framework:

- Preliminary explanation and informed consent: the study’s objective, along with its details, privacy measures, and ethical principles, was presented verbally and in writing to obtain consent.
- Observation during work shift (6 h): participants’ actions were observed during their 6-hour work shift.
- Intervention day: the developed app was used.
- Nonintervention day: the app was not used; participants only carried the device.
- Postshift evaluation: after the work shift, participants answered a questionnaire regarding teamwork perception. The observation of cooperative actions was conducted by observers with nursing qualifications.

### Evaluation Items

This research investigates the clinical impact of app use by evaluating the time taken for responses to occur as its main outcome.

#### Method of Measuring RT

The RT measurement procedure operates identically for both intervention days and nonintervention days. The observer starts timing when the nurse expresses their intention to contact another staff member. The observed period ends when the nurse initiates a work-related dialog with another person, which marks the end of the RT interval.

#### Measurement Scales

The Agency for Healthcare Research and Quality created TeamSTEPPS Teamwork Perceptions Questionnaire (T-TPQ) [10] as a research-based framework for enhancing team performance to improve health care quality and safety [11], and T-TPQ has already been validated in many languages, including Japanese [12-14]. The T-TPQ consists of 35 self-report items that assess individual perceptions about the 5 core competencies: Team Structure, Leadership, Situation Monitoring, Mutual Support, and Communication [15]. This study focused on the Situation Monitoring and Mutual Support competency scales from the T-TPQ because these 2 dimensions are most directly relevant to the intervention mechanism. Situation Monitoring captures nurses’ awareness of their team members’ statuses and the surrounding environment, which is directly facilitated by the app’s real-time broadcast notifications. Mutual Support captures assistance-seeking and assistance-offering behaviors, which are directly targeted by the app’s Green Call and Red Call functions. The remaining 3 T-TPQ competencies (Team Structure, Leadership, and Communication) were considered less directly addressed by this simple signal-based intervention and were therefore excluded from the primary analysis.

### Data Analysis

All statistical analyses were performed using IBM SPSS Statistics and Python (*statsmodels*; Python Software Foundation).

#### Primary Analysis (RT)

The prespecified primary analysis compared per-nurse median RTs between the 15 control and 15 intervention nurses using a 2-sided Mann-Whitney *U* test (exact test). Each nurse’s RT events were first aggregated into a single per-nurse median, yielding the 15-vs-15 comparison and bringing the RT analysis into structural symmetry with the T-TPQ analysis (which is inherently per-nurse).

#### Sensitivity Analyses (RT)

Two prespecified sensitivity analyses on the unaggregated event-level data were performed using linear mixed effects models on log-transformed RT, fitted by restricted maximum likelihood: (model A) log(RT)~Condition+(1 | nurse; Table S1 in Multimedia Appendix 1) and (model B) log(RT)~Condition+(1 | day; Table S2 in Multimedia Appendix 1).

#### Exploratory Analysis (RT by Request Category)

Event-level Mann-Whitney *U* tests were applied within each of the 6 request categories. Per-nurse aggregation within each category was not used because each nurse contributed only 0 to 3 events per category. To control the family-wise error rate across the 6 categories, a Bonferroni correction was applied (adjusted α=.05/6≈.0083).

#### T-TPQ Analysis

The Mann-Whitney *U* test (2-sided, with normal approximation and continuity correction) was applied at the per-nurse level (n=15 per condition) to the total subscale scores for Situation Monitoring and Mutual Support and the 7 individual item scores within each subscale. Subscale-total comparisons were treated as single confirmatory tests and were, therefore, not Bonferroni-adjusted. Item-level tests within each subscale were Bonferroni-adjusted across 7 items (α=.05/7≈.0071).

#### Effect Sizes and Power

Effect sizes were quantified using Cohen *d* on the log scale and rank-biserial *r*. Post hoc power was estimated at α=.05.

#### Sample Size Calculation

To our knowledge, no prior study has evaluated the effect of BLE Mesh–based real-time communication systems on nurse RT or teamwork perceptions. The absence of equivalent preceding research meant that no empirical basis existed for estimating the expected effect size, and accordingly, a formal a priori sample size calculation was not conducted. The study was designed as a feasibility investigation to generate preliminary effect size estimates that can inform the sample size planning of future adequately powered trials. Post hoc power analysis was, therefore, conducted to retrospectively assess whether the achieved sample provided adequate power to detect the observed effects.

### Ethical Considerations

The study was approved by the University of Tsukuba Institutional Review Board (approval 2024R885) and was conducted in accordance with principles equivalent to those of the Declaration of Helsinki. Consent for participation and data use was obtained through an opt-out procedure approved by the institutional review board. A written notice describing the study objectives, the types of data to be recorded, the data protection procedures, and the procedure for declining participation was posted in the ward in advance, and nurses who did not opt out during the notification period were considered to have consented to participation. In addition, prior to each observation session, the research team provided a careful verbal explanation of the study objectives, the data to be recorded, and each participant’s right to decline or withdraw at any time, giving every on-duty nurse repeated opportunities to ask questions or withdraw before the session began. To protect staff privacy, nurse identifiers in the behavioral logs and questionnaire records were replaced with pseudonymous participant IDs at the time of data entry; the linkage table was stored separately on an access-controlled institutional server and was not exported with the analysis files. Communication logs recorded only (1) the time interval between request initiation and response, (2) the request location at the room level (not patient identifier), and (3) the request category; no patient identifiers, patient clinical data, or patient outcomes were recorded. Questionnaire responses were collected in a manner that did not link individual respondents to specific patients. Access to raw logs and questionnaire data was restricted to the research team.

## Results

### Demographic Data

A total of 48 questionnaire responses were collected from 40 unique nurses: 24 responses under the nonintervention condition and 24 under the intervention condition. Because some nurses responded more than once, the following screening steps were applied to ensure unique samples.

First, 5 nurses who participated in both the intervention and nonintervention conditions were excluded to avoid within-subjects contamination, leaving 35 nurses who participated in only 1 condition (18 in the nonintervention group and 17 in the intervention group).

Second, 5 nurses (3 from the nonintervention group and 2 from the intervention group) were excluded because they appeared only as message receivers in the behavioral log and contributed no sender-initiated data, making it impossible to link their questionnaire responses to observed cooperative behaviors.

Finally, 1 nurse, who submitted 2 responses within the nonintervention condition, had the scores averaged to produce a single record.

**Figure 1.**
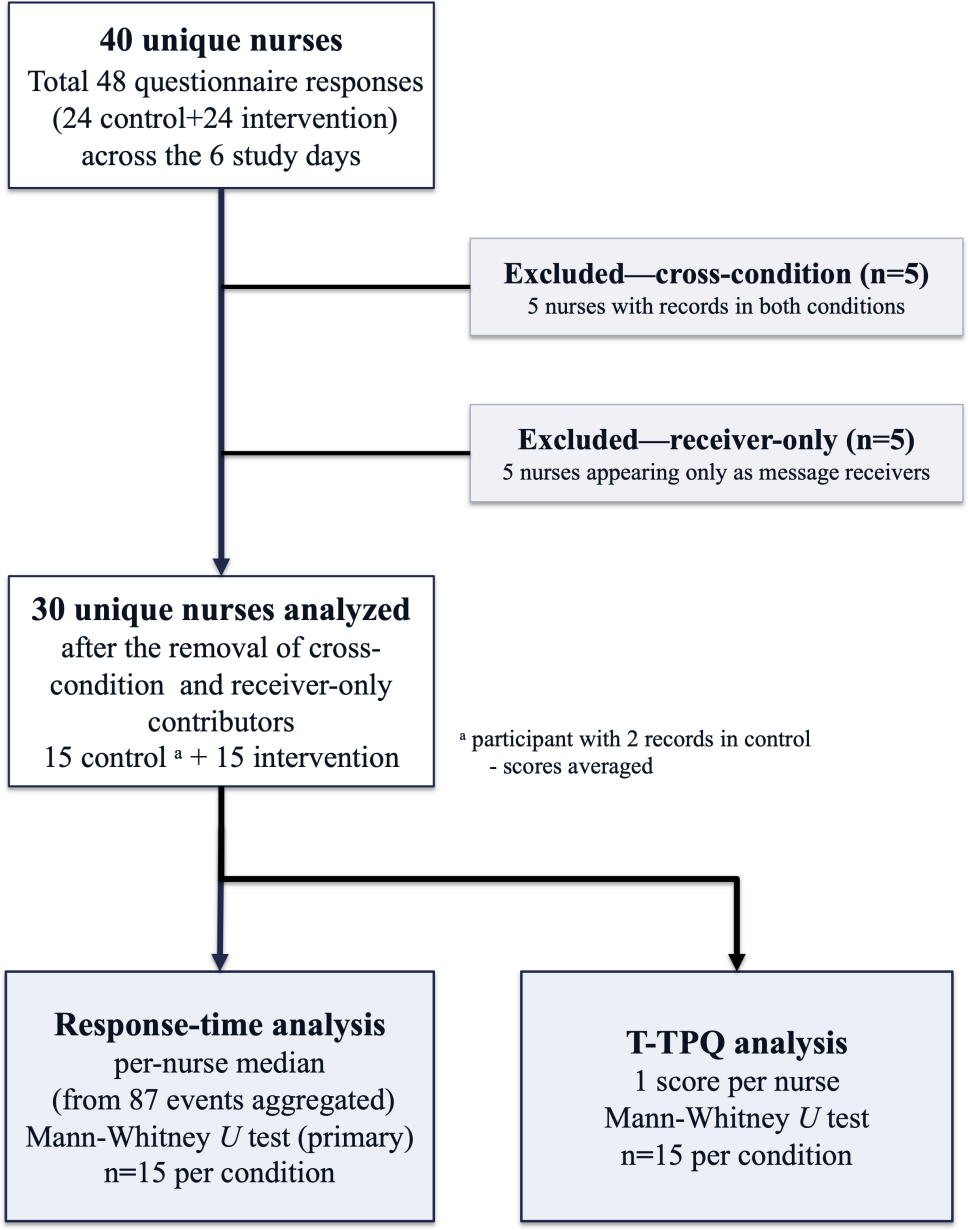
Participant flow diagram. Diagram tracing how 48 questionnaire responses from 40 unique nurses across 6 days of study were reduced to 30 unique participants (15 per condition) for analysis. Exclusions: 5 nurses who provided responses under both conditions, 5 nurses who appeared only as message receivers (with no sender-initiated events), and 1 nurse whose 2 within-condition responses were averaged into a single record. T-TPQ: TeamSTEPPS Teamwork Perceptions Questionnaire.

These steps yielded 15 unique participants per condition (30 total; Figure 1).

### Observed Requests

These 30 participants collectively initiated a total of 87 requests (38 under the control condition and 49 under the intervention condition) across the 6 days of study.

The nonintervention group (control group) made 38 requests, while the intervention group submitted 49 requests. As shown in Table 1, the locations of the requests in the control group (n=38) comprised 13 (34%) requests from 4-bed room, 14 (37%) requests from corridor, 5 (13%) requests from nurse station, 3 (8%) requests from private room, and 3 (8%) requests from day space.

The intervention group (n=49) obtained 22 (45%) requests from the 4-bed room, 12 (24%) requests from corridor, 14 (29%) requests from nurse station, 1 (2%) request from private room, and 0 (0%) requests from day space.

The control group (n=38) included 11 (29%) requests for information sharing, 9 (24%) requests for confirmation to nurse, 8 (21%) requests for care help, 4 (11%) requests for consultation, 4 (11%) requests for inquiry for items, and 2 (5%) requests for confirmation to leader. On the other hand, in the intervention group (n=49), there were 12 (24%) requests for information sharing, 12 (24%) requests for confirmation to nurse, 8 (16%) requests for care help, 11 (22%) requests for consultation, 4 (8%) requests for inquiry for items, and 2 (4%) requests for confirmation to leader.

All observed nurse-initiated requests were classified by the on-site observer into 1 of 6 content categories at the time of each interaction. Care help refers to requests for direct physical assistance with patient care tasks such as repositioning, lifting, or procedural support. Consultation refers to requests for clinical advice or peer consultation regarding patient management decisions. Information sharing refers to the proactive communication of patient-related updates or clinically relevant information to colleagues. Confirmation to nurse refers to requests for verification or acknowledgment from a peer nurse regarding an observation, a procedure, or a care plan. Inquiry for items refers to requests for assistance in locating medical equipment or supplies. Confirmation to leader refers to requests directed to the charge nurse for guidance, approval, or supervisory input.

**Table 1.** Number of requests by category per study condition.

Variable	Control (n=38)	Intervention (n=49)
Count of request, n (%)		
Day 1 (control)	8 (21)	—^a^
Day 2 (intervention)	—	21 (43)
Day 3 (control)	18 (47)	—
Day 4 (intervention)	—	12 (24)
Day 5 (control)	12 (32)	—
Day 6 (intervention)	—	16 (33)
Location of request, n (%)		
4-bed room	13 (34)	22 (45)
Corridor	14 (37)	12 (24)
Nurse station	5 (13)	14 (29)
Private room	3 (8)	1 (2)
Day space	3 (8)	0 (0)
Category of request, n (%)		
Information sharing	11 (29)	12 (24)
Confirmation to nurse	9 (24)	12 (24)
Care help	8 (21)	8 (16)
Consultation	4 (11)	11 (22)
Inquiry for items	4 (11)	4 (8)
Confirmation to leader	2 (5)	2 (4)

a Not applicable.

### Primary Analysis: Per-Nurse RT

**Figure 2.**
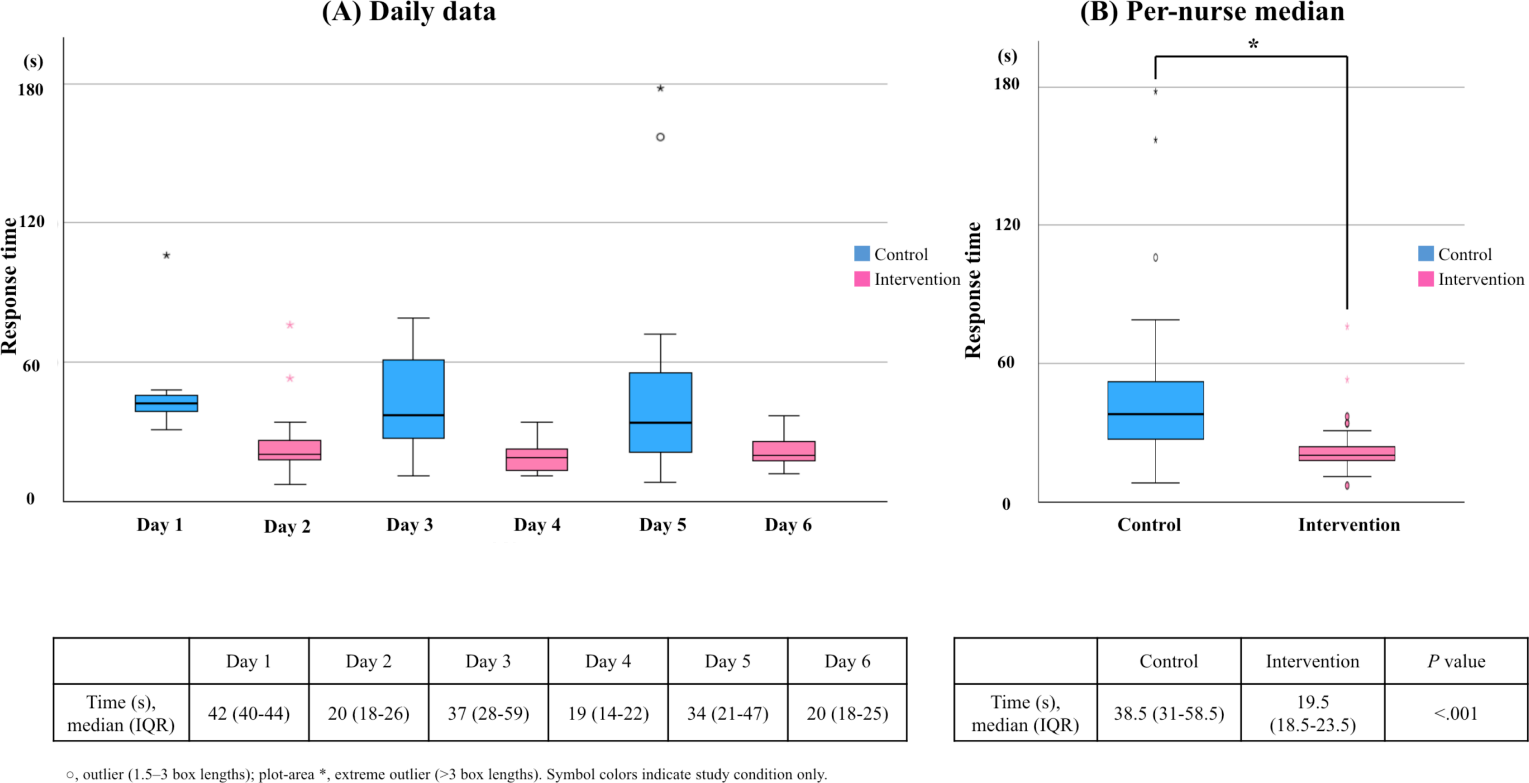
Impact of app use on nurse response times: daily intervention and control data. (A) Event-level response times for each of the 6 study days (boxplots: median, IQR, whiskers) and the corresponding daily medians shown in the accompanying table. (B) Per-nurse median response times for the 15 control and 15 intervention nurses, which constitute the primary analysis; the intervention condition significantly reduced response time (Mann-Whitney *U* test=217.5; *P*<.001; rank-biserial *r*=−0.93, large effect). The asterisk symbol (*) in panel (B) denotes a statistically significant difference between the control and intervention conditions (Mann-Whitney *U* test; *P*<.001).

As the primary analysis, RT observations from the 3-day intervention period and the 3-day nonintervention period were first aggregated to a per-nurse median to account for the within-nurse clustering of repeated observations. The resulting 15 control values and 15 intervention values were then compared using a 2-sided Mann-Whitney *U* test. Daily medians of the unaggregated event-level data are shown in Figure 2 for reference.

The median (IQR) of daily RTs is shown in Figure 2 as follows: day 1 (control) was 42 (IQR 40-44) seconds, day 2 (intervention) was 20 (IQR 18-26) seconds, day 3 (control) was 37 (IQR 28-59) seconds, day 4 (intervention) was 19 (IQR 14-22) seconds, day 5 (control) was 34 (IQR 21-47) seconds, and day 6 (intervention) was 20 (IQR 18-25) seconds.

At the per-nurse level, the median RT was 38.5 (IQR 31.0-58.5) seconds in the control condition and 19.5 (IQR 18.5-23.5) seconds in the intervention condition. The difference was statistically significant (Mann-Whitney *U* test=217.5; *P*<.001; rank-biserial *r*=–0.93, large effect).

### Sensitivity Analysis: Linear Mixed Effects Model

To formally account for the within-nurse clustering of repeated observations, a prespecified sensitivity analysis fitted a linear mixed effects model to all 87 events with log-transformed RT as the outcome, condition as a fixed effect, and a random intercept for nurse (Table S1 in Multimedia Appendix 1). The intervention reduced log-transformed RT by β of −0.67 (95% CI −0.93 to −0.41; *P*<.001), corresponding to a 48.7% reduction in geometric mean RT. The within-nurse intraclass correlation of 0.14 was modest, indicating that the per-nurse aggregation used in the primary analysis provides an interpretable summary, while the mixed effects model formally confirms the result under the residual within-nurse correlation.

### Exploratory Analysis: RT by Request Category

**Figure 3.**
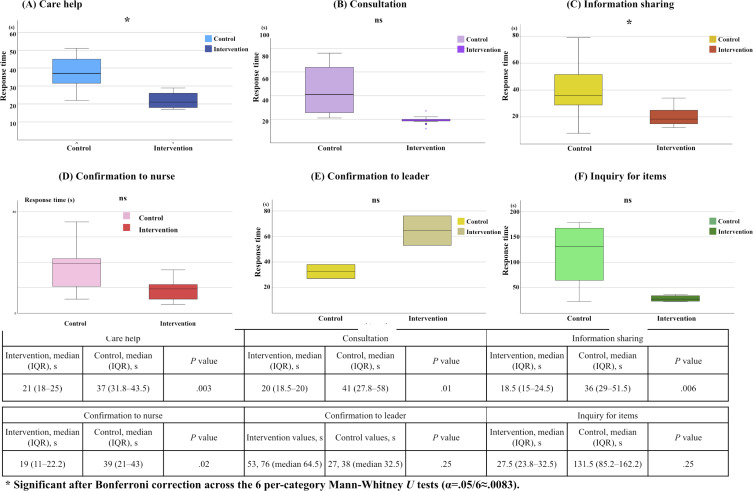
Impact of app use for category of request. Boxplots show the median response time (IQR values) for each of the 6 request categories under the control and intervention conditions, with per-category Mann-Whitney *U* test values and *P* values listed in the accompanying tables. Bonferroni correction was applied across the 6 categories (adjusted α=.05/6≈.0083). Care help (*P*=.003) and information sharing (*P*=.006) remained significant after correction; consultation (*P*=.01) and confirmation to nurse (*P*=.02) showed nominal reductions that did not survive correction; Inquiry for items (*P*=.25) and confirmation to leader (*P*=.25) did not differ between conditions. ns: not significant.

As an exploratory analysis, event-level RTs were compared between conditions within each of the 6 request categories using 2-sided Mann-Whitney *U* tests (Figure 3). To control the family-wise error rate across the 6 categories, Bonferroni correction was applied (adjusted α=.05/6≈.0083). Two categories remained significant after correction: “Care help” decreased from 37 (control) to 21 seconds (intervention; *P*=.003) and “Information sharing” decreased from 36 to 18.5 seconds (*P*=.006). Two further categories showed nominal reductions of similar magnitude but did not survive Bonferroni correction: “Consultation” (41-20 s; *P*=.01) and “Confirmation to nurse” (39-19 s; *P*=.02). “Inquiry for items” showed a numerically large reduction from 131.5 to 27.5 seconds that did not reach statistical significance (*P*=.25). For “Confirmation to leader,” median values were 32.5 seconds (control; n=2) and 64.5 seconds (intervention; n=2); with only 2 observations per condition, this comparison was underpowered (*P*=.25) and should be interpreted with caution.

### Effect on Perceived Situation Monitoring (T-TPQ)

**Figure 4.**
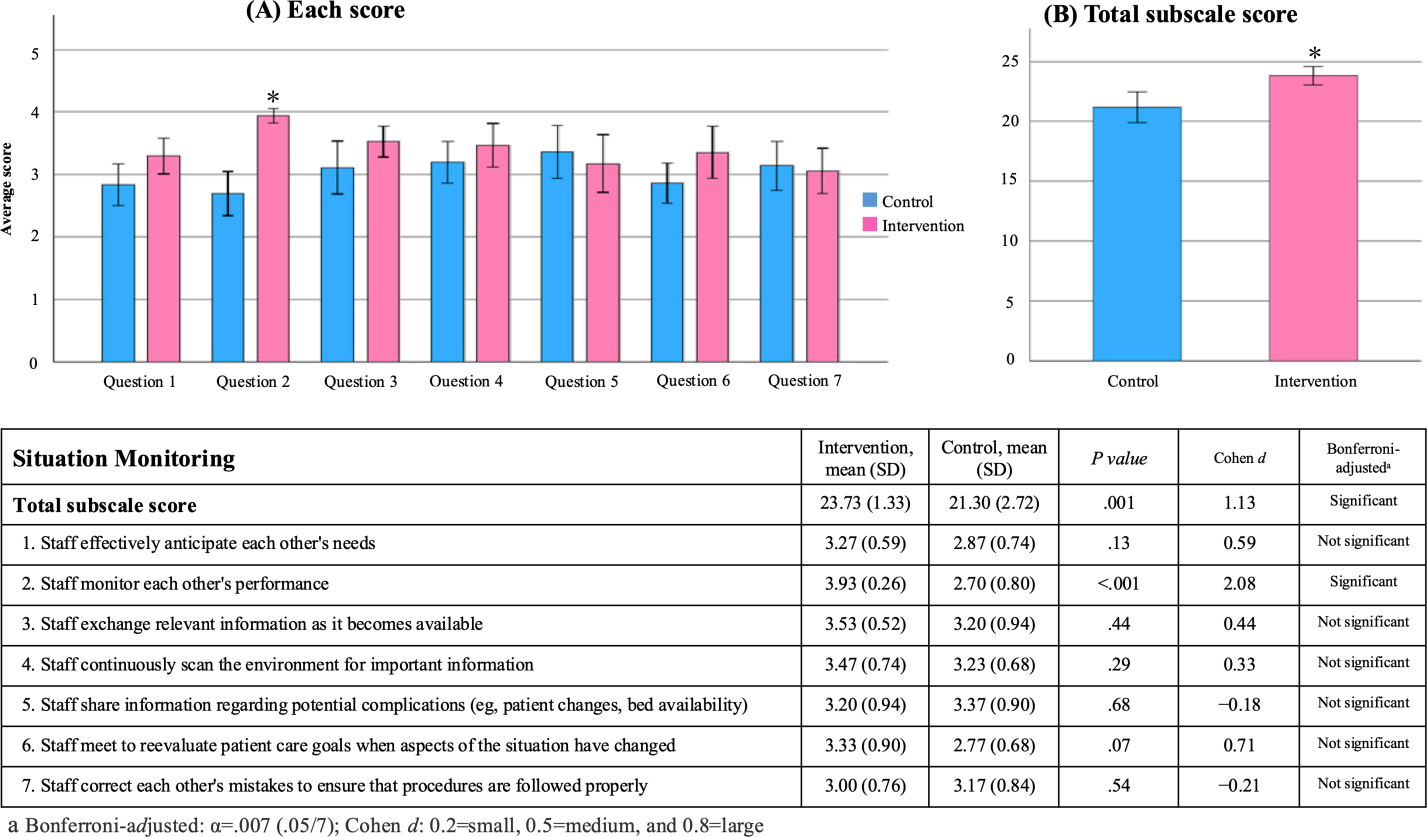
Impact of the intervention on perceived Situation Monitoring (TeamSTEPPS Teamwork Perceptions Questionnaire domain). (A) Average score for each of the 7 Situation Monitoring items (Question 1-Question 7) under the control and intervention conditions (error bars represent the SE). (B) Total subscale score (sum across Question 1-Question 7). Item-level Mann-Whitney *U* tests were Bonferroni-adjusted across the 7 items (α=.05/7≈.0071); the subscale-total comparison was a single confirmatory test and was, therefore, not Bonferroni-adjusted. The total subscale score improved significantly under the intervention condition (3.04 to 3.39; *P*=.001; Cohen *d*=1.13, large effect). At the item level, Question 2 (“Staff monitor each other’s performance”) was the only item that survived Bonferroni correction (*P*<.001). Asterisks (*) denote items significant comparisons: in panel (A), the item that survived Bonferroni correction (question 2; *P*<.001; Bonferroni-adjusted α=.05/7≈.0071); in panel (B), the significant confirmatory subscale-total comparison (*P*=.001; not Bonferroni-adjusted, as prespecified).

T-TPQ results were analyzed using the Mann-Whitney *U* test on 30 unique participants (n=15 per condition) identified from sender activity logs. For Situation Monitoring, the Mann-Whitney *U* test was applied to check for significant differences between individual T-TPQ items, with a Bonferroni-adjusted α of .0071. As shown in Figure 4, 1 item reached statistical significance after correction: question 2, “Staff monitor each other’s performance” increased from 2.70 (control) to 3.93 (intervention); *P*<.001 (significant, large effect). The remaining 6 items did not reach significance: question 1, “Staff effectively anticipate each other’s needs” (control 2.87 vs intervention 3.27; *P*=.13); question 3, “Staff exchange relevant information as it becomes available” (3.20 vs 3.53; *P*=.44); question 4, “Staff continuously scan the environment for important information” (*P*=.29); question 5, “Staff share information regarding potential complications (eg, patient changes, bed availability)” (3.37 vs 3.20; *P*=.68); question 6, “Staff meet to reevaluate patient care goals when aspects of the situation have changed” (2.77 vs 3.33; *P*=.07); and question 7, “Staff correct each other’s mistakes to ensure that procedures are followed properly” (3.17 vs 3.00; *P*=.54). The Situation Monitoring total score improved from a mean of 3.04 (SD 0.39) in the control group to 3.39 (SD 0.19) in the intervention group (Figure 4), and the difference was statistically significant (*P*=.001; Cohen *d*=1.13, large effect).

### Effect on Perceived Mutual Support (T-TPQ)

**Figure 5.**
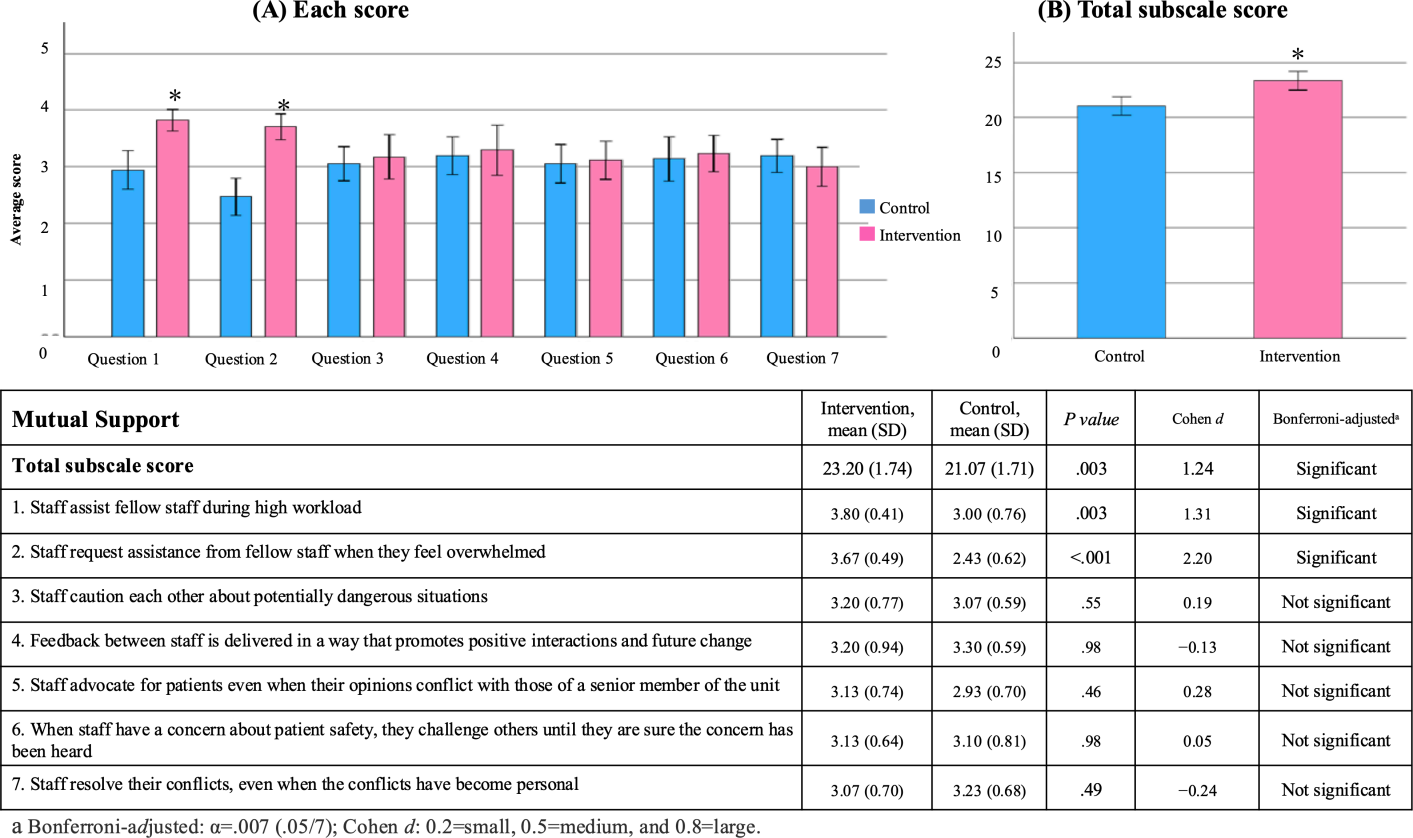
Impact of the intervention on perceived Mutual Support (TeamSTEPPS Teamwork Perceptions Questionnaire domain). (A) Average score for each of the 7 Mutual Support items (Question 1-Question 7) under the control and intervention conditions (error bars represent the SE). (B) Total subscale score (sum across Question 1-Question 7). Item-level Mann-Whitney *U* tests were Bonferroni-adjusted across the 7 items (α=.05/7≈.0071); the subscale-total comparison was a single confirmatory test and was, therefore, not Bonferroni-adjusted. The total subscale score improved significantly under the intervention condition (3.01 to 3.31; *P*=.003; Cohen *d*=1.24, large effect). At the item level, 2 items survived Bonferroni correction: Question 1 (“Staff assist fellow staff during high workload”; *P*=.003) and Question 2 (“Staff request assistance from fellow staff when they feel overwhelmed”; *P*<.001). Asterisks denote items significant comparisons: in panel (A), the items that survived Bonferroni correction (question 1, *P*=.003; question 2, *P*<.001; Bonferroni-adjusted α=.05/7≈.0071); in panel (B), the significant confirmatory subscale-total comparison (*P*=.003).

As shown in Figure 5, for Mutual Support, 2 items reached statistical significance after Bonferroni correction: question 1, “Staff assist fellow staff during high workload,” increased from 3.00 (control) to 3.80 (intervention), *P*=.003 (significant); question 2, “Staff request assistance from fellow staff when they feel overwhelmed,” increased from 2.43 to 3.67, *P*<.001 (significant, large effect). The remaining 5 items did not reach significance: question 3, “Staff caution each other about potentially dangerous situations” (3.07 vs 3.20; *P*=.55); question 4, “Feedback between staff is delivered in a way that promotes positive interactions and future change” (3.30 vs 3.20; *P*=.98); question 5, “Staff advocate for patients even when their opinions conflict with those of a senior member” (2.93 vs 3.13; *P*=.46); question 6, “When staff have a concern about patient safety, they challenge others until they are sure the concern has been heard” (3.10 vs 3.13; *P*=.98); and question 7, “Staff resolve their conflicts, even when the conflicts have become personal” (3.23 vs 3.07; *P*=.49). The Mutual Support total score improved from a mean of 3.01 (SD 0.24) in the control group to 3.31 (SD 0.25) in the intervention group, as shown in Figure 5, and the difference was highly significant (*P*=.003; Cohen *d*=1.24, large effect).

## Discussion

### Effects on Teamwork Perceptions (T-TPQ)

This intervention study was conducted with adequate statistical power across all primary outcomes. Post hoc power analyses confirmed that the achieved sample yielded 90% power for the overall RT outcome (Cohen *d*=1.19), 87% power for Situation Monitoring (Cohen *d*=1.13), and 92% power for Mutual Support (Cohen *d*=1.24), all exceeding the conventional 80% threshold. The findings discussed below are, therefore, considered robust within the scope of this single-site intervention study.

The minimal interaction communication app developed in this study, which uses BLE Mesh network technology, has been shown to be extremely effective in streamlining communication and improving teamwork among nurses. The intervention resulted in a significant reduction in overall RT and a significant improvement in the T-TPQ scores for both Situation Monitoring and Mutual Support. These results strongly suggest that this app can be a practical solution to communication challenges in the nursing field.

The most significant factor in the overall reduction in RT in this study is considered to be the transformation of the communication style itself: from making voice calls to specific individuals to being able to send a brief help signal to the whole team at once, without speaking, when assistance is needed. This way of sending a quick signal to the whole team without speaking streamlined operations through different mechanisms, depending on the nature of the task.

Among the 6 request categories, the most robust improvements—those that survived Bonferroni correction across the 6 per-category comparisons (adjusted α=.0083)—were observed for “Care help” (37-1 s; *P*=.003) and “Information sharing” (36-18.5 s; *P*=.006). For “Care help,” assistance can typically be rendered by any available nurse with the requisite physical capacity, and sending the same signal to the whole team at once enabled instant access to the team’s collective availability, reducing the time cost of locating a willing helper. For “Information sharing,” sending the same signal to the whole team at once allowed clinically relevant updates to reach the team without interrupting the work of a specific recipient: a single signal reaches every team member at once, rather than being relayed from 1 nurse to the other.

Two additional categories—“Consultation” (41-20 s; *P*=.01) and “Confirmation to nurse” (39-19 s; *P*=.02)—showed nominal reductions of comparable magnitude but did not survive Bonferroni correction for multiple comparisons across the 6 categories. These results are best interpreted as hypothesis-generating: they are directionally consistent with the broadcast mechanism described above (ie, requests that can be fulfilled by any qualified peer rather than by a specific individual), but the present feasibility sample lacks the statistical resolution to confirm independent effects in these subcategories. The nonsignificant change in “Inquiry for Items” (131.5-27.5 s; *P*=.25), despite a numerically large median reduction, is likewise underpowered owing to the small per-category sample (n=4 per condition).

On the other hand, the limitations of this model were also revealed by the results for “Confirmation to leader.” RT did not improve in this category alone because the request was directed to a specific individual in the team who had an irreplaceable role. In this case, the issue was not the time it took to find the leader but securing the leader’s availability to respond, a problem that could not be solved by a simple broadcast. This is related to the busyness and digital stress of leadership positions, as pointed out by Laukka et al [16], and suggests that the technology to be introduced should be optimized according to the nature of the problem to be solved.

Overall, these category-level patterns are consistent with the broadcast mechanism described above.

The T-TPQ served as the assessment tool to measure teamwork perception changes, which showed substantial improvements in both the Situation Monitoring and Mutual Support categories. The app’s real-time notification system, which makes each staff member’s current situation visible to the rest of the team, likely caused the improvement in Situation Monitoring scores by giving every nurse the same picture of where each colleague is and what each is doing. The Mutual Support score improvement stands as a crucial discovery from this research. The literature shows that measuring Mutual Support in nursing teams becomes difficult because of the difference between staff-provided assistance and staff-received assistance. The app successfully reduced psychological barriers to help requests, according to our findings, which show substantial improvements in “Staff assist fellow staff during high workload” and “Staff request assistance from fellow staff when they feel overwhelmed.” The decreased barriers enabled staff members to request help without hesitation, while available staff members provided support voluntarily, thus solving the problem identified in previous studies [17].

On the other hand, no significant difference was observed in items requiring more complex and advanced communication, such as “Staff caution each other about potentially dangerous situations” and “Staff resolve their conflicts.” This indicates that while the app contributes to providing an “impetus” for and “streamlining” communication, it does not guarantee the content or quality of the interaction itself. Among individual Situation Monitoring items, question 1 (“Staff effectively anticipate each other’s needs”) and question 3 (“Staff exchange relevant information as it becomes available”) did not reach statistical significance (*P*=.13 and *P*=.44, respectively). Critically, the overall subscale totals for both Situation Monitoring and Mutual Support were significant (*P*=.001 and *P*=.003, respectively), with large effect sizes (Cohen *d*=1.13 and 1.24), supporting the robustness of the primary findings.

Previous research shows that even a simple, content-light signal can convey complex and rich meaning when senders and receivers share an existing working relationship, along with the social, cultural, and emotional context that comes with it [18].

This experiment used these brief team-wide signals—sent without speaking, to the whole team at once—as an example of how a short, content-light signal can still convey rich meaning when the people involved share the clinical context. Because senders and receivers already share the same ward context, a short signal can carry several layers of meaning, and meaningful cooperative behavior becomes possible with very little explicit content. The results show that what matters is not the amount of information carried by the signal, but the depth of interpretation that the professionals on each end of the signal bring to it.

Minimal communication should, therefore, be viewed not as a limitation but as a starting point that draws on the nurses’ interpretive skills, builds on their working relationships, and uses their clinical experience to produce a shared understanding.

The result of this study suggests that successful outcome is determined not only by the superiority of the technology itself but also by how it is accepted and used in the field. Livesay et al [19] point out that a major reason for the failure of digital health adoption is the lack of nurse involvement in the design and implementation process, as well as inadequate training, indicating that technology lacking a field perspective can disrupt workflows. Furthermore, Fitzpatrick [20] warns of the overlooked negative aspect of digitalization, where the increase in communication volume can, conversely, contribute to health care provider burnout. Additionally, the adoption rate among staff can be an issue in this type of technology implementation. For example, in a contact tracing study by Curtis et al [21], the participation rate, especially among physicians, was reported to be low despite the use of anonymous tags, possibly due to privacy concerns. The key to the success of these technological innovations lies in the close collaboration between nurses, who have a deep understanding of the nursing field, and researchers and developers in the engineering field. Wang et al [22] emphasize that deep collaboration between nursing professionals and Internet of Things technology developers is essential for the effective implementation of these technologies. It should also be acknowledged that this study evaluated teamwork perceptions using only 2 of the 5 T-TPQ subscales, and that self-report questionnaire data had inherent limitations in capturing the full complexity of team processes. Perceptual scores reflect nurses’ subjective appraisals and may be susceptible to response bias or social desirability effects. Objective behavioral measures of teamwork, such as structured observational coding or interaction analysis, would complement the self-report data in future studies. Furthermore, the 3 T-TPQ subscales not assessed in this study (Team Structure, Leadership, and Communication) may also have been influenced by the intervention in ways not captured here, and future research should consider comprehensive multidimensional teamwork assessments. Only when digital health is built on the needs of the clinical floor and keeps the nurses who actually use it firmly in view can it achieve its ultimate goal of improving the quality and safety of health care [19-25].

### Limitations

This study has several limitations, including a small sample size of 8 nurses per shift at a single acute care ward; a short study period of 6 days, which requires caution in generalizing the results; the lack of verification of the Red Call function for emergencies; and the fact that the evaluation was primarily quantitative. For RT data, each nurse could contribute multiple events across multiple days. The primary analysis addressed this by aggregating events to a per-nurse median before testing (n=15 per condition), and a linear mixed effects model was fitted as a sensitivity check; both analyses yielded congruent results. The per-category RT analyses, however, retained the event-level unit of analysis because each nurse contributed too few events per category for per-nurse aggregation within a category to be informative. Per-category *P* values were corrected for multiplicity across the 6 request categories using Bonferroni adjustment (adjusted α=.0083); 2 categories (Care Help and Information Sharing) remained significant after correction, while 2 further categories (Consultation and Confirmation to Nurse) showed nominal reductions that did not survive correction and should be regarded as hypothesis-generating. Additionally, the alternating-day, between-subjects design is susceptible to temporal confounding from factors, such as staffing variation, patient census, and workload differences between study days. Although day-level clustering was examined in a prespecified sensitivity analysis (Table S2 in Multimedia Appendix 1) and did not alter the substantive conclusion, day-level confounders cannot be fully excluded with the present design and require confirmation in a larger study with a greater number of study days.

### Conclusions

This study demonstrated that a simple signal communication app using BLE Mesh network technology significantly reduces RTs among nurses and markedly improves the core teamwork elements of Situation Monitoring and Mutual Support. This indicates that the app has immediate and practical value in improving the inefficiency of communication in nursing practice and enhancing the quality of team-based medical care.

## Supplementary material

10.2196/85599 Multimedia Appendix 1 Supplementary tables for the linear mixed-effects sensitivity analyses.

## Abbreviations

BLE: Bluetooth Low Energy
RT: response time
T-TPQ: TeamSTEPPS Teamwork Perceptions Questionnaire
