# Supplementary material for: Facilitating Teamwork With Minimal Interaction via a Mesh-Based Communication Device in a Clinical Nursing Setting: Intervention Study
Source: JMIR Nurs. 2026 Aug 5;9:e85599. doi: 10.2196/85599 (PMC13447815; doi:10.2196/85599)
Supplement: Supplementary file 1 [file nursing-v9-e85599-s001.docx]

**Supplementary Table S1. Linear mixed-effects model — Model A: random intercept for nurse.**

Outcome: log-transformed response time. Fixed effect: Condition (Intervention = 1, Control = 0). Random effect: random intercept for nurse. Sample: n = 87 events from 30 nurses (15 per condition). Estimation: restricted maximum likelihood (REML); software: Python statsmodels.

| **Parameter** | **Estimate** | **SE** | **z** | **95% CI** | **p** |
| --- | --- | --- | --- | --- | --- |
| Intercept (Control log-RT) | 3.677 | 0.097 | 37.99 | [3.49, 3.87] | < .001 |
| Intervention (vs Control) | −0.668 | 0.133 | −5.02 | [−0.93, −0.41] | < .001 |
| Random intercept variance (nurse) | 0.038 | — | — | — | — |
| Residual variance | 0.230 | — | — | — | — |
| Within-nurse ICC | 0.14 | — | — | — | — |

**Notes. The fixed effect of Condition corresponds to a multiplicative effect of exp(−0.668) = 0.513 on the original (seconds) scale, i.e., a 48.7% reduction in geometric mean response time under the intervention condition. ICC indicates modest within-nurse clustering that is formally accounted for by the random intercept.**

**Supplementary Table S2. Linear mixed-effects model — Model B: random intercept for study day.**

Outcome: log-transformed response time. Fixed effect: Condition (Intervention = 1, Control = 0). Random effect: random intercept for study day (n = 6 days, 3 per condition). Sample: n = 87 events. Estimation: REML.

| **Parameter** | **Estimate** | **SE** | **z** | **95% CI** | **p** |
| --- | --- | --- | --- | --- | --- |
| Intercept (Control log-RT) | 3.652 | 0.062 | 58.6 | [3.53, 3.77] | < .001 |
| Intervention (vs Control) | −0.626 | 0.101 | −6.21 | [−0.82, −0.43] | < .001 |
| Random intercept variance (day) | 0.000 | — | — | — | — |
| Residual variance | 0.266 | — | — | — | — |
| Day-level ICC | 0.000 | — | — | — | — |

**Notes. The Day random intercept variance estimated at the boundary (≈ 0; singular fit), indicating that day-level variability beyond what is explained by Condition is negligible. The Condition fixed effect is consistent in direction and magnitude with Model A (−0.626 vs −0.668; both p < .001), corresponding to a 46.5% reduction in geometric mean response time. Together with Model A, this confirms that the conclusion is robust to whether clustering is modelled at the nurse level or the day level.**
